# A critical evaluation of dynamical systems models of bipolar disorder

**DOI:** 10.1038/s41398-022-02194-4

**Published:** 2022-09-28

**Authors:** Abraham Nunes, Selena Singh, Jared Allman, Suzanna Becker, Abigail Ortiz, Thomas Trappenberg, Martin Alda

**Affiliations:** 1 Department of Psychiatry, Dalhousie University, Halifax, NS Canada; 2 Faculty of Computer Science, Dalhousie University, Halifax, NS Canada; 3 Department of Psychology, Neuroscience & Behaviour, McMaster University, Hamilton, ON Canada; 4 Department of Psychiatry, University of Toronto, Toronto, ON Canada; 5 Centre for Addiction & Mental Health, Toronto, ON Canada

**Keywords:** Bipolar disorder, Human behaviour

## Abstract

Bipolar disorder (BD) is a mood disorder involving recurring (hypo)manic and depressive episodes. The inherently temporal nature of BD has inspired its conceptualization using dynamical systems theory, which is a mathematical framework for understanding systems that evolve over time. In this paper, we provide a critical review of the dynamical systems models of BD. Owing to the heterogeneity of methodological and experimental designs in computational modeling, we designed a structured approach that parallels the appraisal of animal models by their face, predictive, and construct validity. This tool, the validity appraisal guide for computational models (VAG-CM), is not an absolute measure of validity, but rather a guide for a more objective appraisal of models in this review. We identified 26 studies published before November 18, 2021 that proposed generative dynamical systems models of time-varying signals in BD. Two raters independently applied the VAG-CM to the included studies, obtaining a mean Cohen’s *κ* of 0.55 (95% CI [0.45, 0.64]) prior to establishing consensus ratings. Consensus VAG-CM ratings revealed three model/study clusters: data-driven models with face validity, theory-driven models with predictive validity, and theory-driven models lacking all forms of validity. We conclude that future modeling studies should employ a hybrid approach that first operationalizes BD features of interest using empirical data to achieve face validity, followed by explanations of those features using generative models with components that are homologous to physiological or psychological systems involved in BD, to achieve construct validity. Such models would be best developed alongside long-term prospective cohort studies involving a collection of multimodal time-series data. We also encourage future studies to extend, modify, and evaluate the VAG-CM approach for a wider breadth of computational modeling studies and psychiatric disorders.

## Introduction

Bipolar disorder (BD) is a mood disorder of unknown etiology characterized by episodes of mania, depression, and periods of euthymia [1–5]. Many studies have investigated BD during episodes of mania, depression, or euthymia, but fewer have studied the time-course of transitions and episodicity of activity, cognition, and emotion. This longitudinal course is a critical element for understanding BD [6], and is predictive of treatment response [7].

Dynamical systems theory is a mathematical framework for studying systems that evolve over time (reviewed in Supplemental Materials and [8, 9]), which makes it an appealing framework for studying and modeling time-varying aspects of BD. A dynamical systems model captures the relationship between a system’s features of interest and an “evolution rule” that describes how those features change over time. Sets of differential equations (in the continuous time setting) or difference equations (in the discrete-time setting) are used to describe these relationships. Researchers can decide to use ordinary (deterministic) or stochastic equations depending on whether the system being modeled exhibits randomness or noise in its evolution. For instance, the random noise component in a stochastic model of mood dynamics in BD may represent fluctuations in an individual’s environment [10, 11]. Dynamical systems modeling approaches can therefore be used to mathematically conceptualize theoretical mechanisms of BD, such as those linking affective dysfunction to abnormalities of the behavioral activation system [12], reinforcement learning [13–16] and the neuroanatomical circuits governing emotional processing [17] or circadian function [18, 19]. Such mathematical representations should force models’ assumptions and predictions to be identified clearly. Furthermore, competing mechanistic hypotheses can be represented using different model architectures, and their relative explanatory power can be evaluated with statistically rigorous model selection procedures [20]. The use of these dynamical systems methods in BD research dates back more than 20 years [21], but their contributions to the understanding of BD are unclear.

While studies using dynamic systems models of BD may explain known phenomena and generate testable predictions for further research, we currently lack standardized approaches to critically appraise their validity. A preliminary review of this literature reveals marked heterogeneity in the target audiences, technical complexity, and clinical relevance. We, therefore, sought to appraise these studies in a structured and transparent way by introducing a set of appraisal criteria that mirror criteria for validating animal models in psychiatry. While this approach was designed specifically for the present study, we kept it sufficiently general to facilitate re-use, extension, and evaluation by researchers interested in other conditions or models. Using this structured approach, the present review will address the face, predictive, and construct validity of dynamical systems models of BD, and summarize the insights they have provided about its temporal course and mechanisms. We then highlight conceptual and methodological gaps in this literature, and propose a roadmap for further computational modeling studies of BD.

## Methods

### Search strategy and evaluation

The Scopus database was searched from inception until November 18, 2021 using the following search query:

TITLE-ABS-KEY((((affective OR bipolar) AND disorder) OR “manic depression” OR “manic depressive” OR antidepressant) AND (“mood dynamics” OR “mood variability” OR “mood variations” OR circadian OR “biological rhythms”) AND (((bistability OR multistability) OR (chaos OR “chaos theory” OR “strange attractor”) OR (“lienard oscillators” OR “limit cycle” OR “limit cycle oscillators”) OR (“nonlinear dynamics” OR “oscillations” OR “perturbation method” OR “recurrent map” OR “stochastic resonance” OR “winnerless competition”) OR (“crisis” OR “critical slowing down” OR intermittency OR “chaotic intermittency”)) OR (“computational modeling” OR “mathematical modeling” OR “time-series analysis” OR “mechacognition” OR “cognitive network”))).

This query is split into three parts, requiring that papers touch on all of (A) bipolar disorder, (B) mood or circadian dynamics, and (C) dynamical systems. Within each of these parts, additional terms are included using OR operators. Since papers on this topic are published across different scientific fields, we iteratively built this query using keywords from relevant search results (using OR operators) until the number of results converged on a stable value. This was done to minimize the risk of missing work in other disciplines by virtue of nomenclature differences.

We included studies that have (A) been published in English language peer-reviewed journals and (B) proposed generative dynamical systems models of mood, activation, circadian rhythm fluctuation, or other time-series signals in BD. We focused on generative models because they do not simply describe some phenomenon, but can also *produce it* through simulation. Reference lists of included manuscripts were subsequently searched for papers that may have been missed by our initial search. We also searched lists of papers that cited the included studies for further papers that may have been missed.

### Evaluation of model quality

**Fig. 1:**
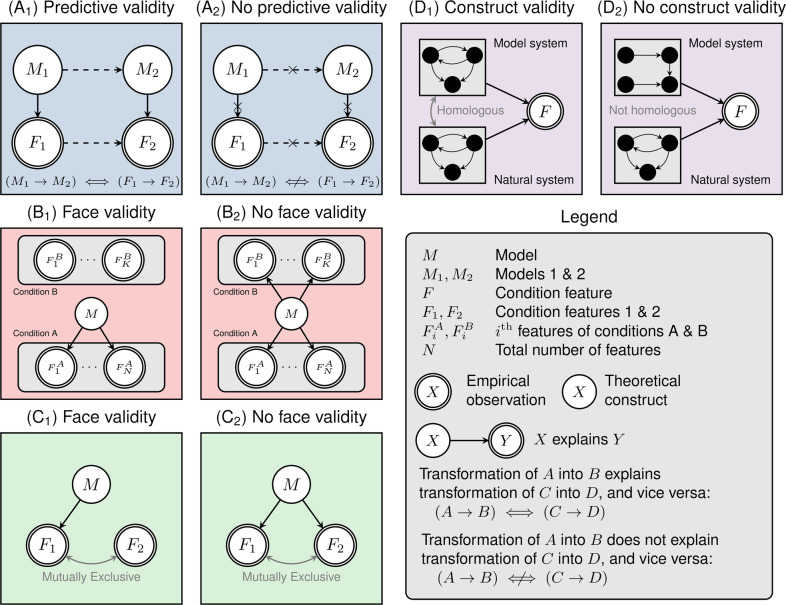
Illustration of the validity appraisal guide for computational models of psychiatric disorders. Abbreviations and symbols are shown in the legend panel. Predictive validity (Panels A_1_ and A_2_): The presence of predictive validity requires identifying distinct features (here *F*_*1*_ and *F*_*2*_), each of which is specifically explained by distinct models *M*_*1*_ and *M*_*2*_. One must show that there exists a real-world transition (such as medication use) that results in the transition from feature *F*_*1*_ to *F*_*2*_, and that this can be adequately modeled by a transformation of model *M*_*1*_ into *M*_*2*_. Face validity (Panels B_1_, B_2_, C_1_, C_2_): To establish face validity, one must identify features that characterize a target condition (such as bipolar disorder; here *Condition A*), denoted *F*_*1*_^*A*^*, F*_*2*_^*A*^*,* *…* *, F*_*N*_^*A*^. Ideally, features that characterize a relevant comparator, *Condition B*, should also be identified (*F*_*1*_^*B*^*, F*_*2*_^*B*^*,* *…* *, F*_*K*_^*B*^). If model *M* has face validity, then it should be able to explain as many features of condition A as possible, while not explaining features of condition B. Finally, if model *M* explains some feature *F*_*1*_, then it should *not* explain mutually exclusive features *F*_*2*_. Construct validity (Panels D_1_, D_2_): To establish construct validity, one must identify the components of a natural system, such as a biochemical pathway or neural circuit, and establish that the functioning of that system explains some feature(s) *F*. A model system has construct validity if it is specified at a level of abstraction such that individual components and interactions are homologous to those present in the natural system.

To evaluate computational models, we devised a set of criteria analogous to those used for validating animal models of neuropsychiatric disorders [22]. These criteria were divided into checklists concerning face validity, predictive validity, and construct validity, respectively (Fig. 1). A full copy of this validity appraisal guide for computational models (VAG-CM) is included in the Supplemental Materials.

#### Validity appraisal guide for computational models (VAG-CM)

After previously reviewing several manuscripts on dynamical systems models of BD, one author (AN) found that harmonizing their quality and findings was difficult without a clear structured approach. As such, the VAG-CM was developed in order to better organize this review. The VAG-CM is not intended as an absolute evaluation of model validity, but rather as a structured approach to appraise BD studies employing computational models. Although the VAG-CM was specifically created for this review, we attempted to design it with sufficient generality so other researchers could potentially apply or extend it to other domains and computational model types, such as those of neurons, circuits, or cognitive mechanisms [23–25]. By adapting an approach and nomenclature familiar to the psychiatric basic science community, we aimed for the VAG-CM to facilitate successful translation and interplay between computational modelers and experimental psychiatric researchers. While there are other disciplines, such as philosophy of science, which are concerned with the appraisal of model quality in neuroscience, we are not aware of investigations producing a structured appraisal approach for reviews such as this. The approach used in animal modeling literature was also selected because it (A) has been developed for the purposes of establishing biological models of practical consequence for managing psychiatric disorders in humans [22], (B) prioritizes pragmatic utility over philosophical abstractions, and (C) highlights the necessary ties that must be built between experimental and computational modeling researchers.

The VAG-CM attempted to minimize the influence of reviewers’ subjective impressions of the merits of a model’s assumptions. For example, consider a model that makes the following simple but non-trivial assumption: that “mood” at time *t* is independent of mood at time *t-1* (i.e., a Markovian assumption). The VAG-CM required reviewers to simply identify whether study authors offered theoretical or empirical justification (with appropriate citations) for that assumption. This was done to limit the degree to which reviewers judged assumptions based on their a priori knowledge.

The first subscale, concerning face validity, asks whether a real-world phenomenon has been sufficiently (A) characterized and then (B) explained by a model (Fig. 1). For a BD-related phenomenon to be sufficiently characterized, modelers must provide a robust theoretical or empirical justification of their assumptions based on the analysis of raw data or through the presentation of a clearly documented literature review. When evaluating a modeling study using the VAG-CM, the reviewer places the responsibility on the modeling study to justify that the phenomenon modeled is relevant, operationalized, and empirically identifiable.

In this subscale, we have also tried to capture evidence of convergent and divergent validity. Convergent validity is the degree to which a model of some condition can describe multiple features of that condition. Divergent validity requires that the model can explain features of its target condition, while not being able to capture features of some relevant comparator condition.

#### Face validity

The degree to which the model exhibits behaviors similar to the condition of interest.

- The model describes a real-world phenomenon (i.e., a target state/condition vis a vis comparators).
- The target state/condition being modeled is identifiable according to observable features.
- The comparator state/condition being modeled is identifiable according to observable features.
- The model actually explains/predicts the target condition vis a vis the comparator.

The second subscale, concerning predictive validity, asks whether outputs from simulated interventions on the model change in a fashion that mirrors the effects of some real-world intervention (Fig. 1). Furthermore, this subscale asks if the intervention on the model is empirically or theoretically justified as a representation of a real-world change to the system. For example, if model *M*_1_ captures depression (*D*), and model *M*_2_ captures normal mood (euthymia, *E*), then one may identify a real-world transition resulting in *D* → *E* mediated by antidepressants or mood-stabilizers (or even just the passage of time). One could then model that transformation as some function that turns *M*_1_ → *M*_2_ (thereby resulting in a shift from *D* → *E* in the model output). Such a model would be a candidate for predictive validity, since the form and consequences of the transformed model could be tested empirically. Note that predictive validity is not solely applicable to treatment-related phenomena. For example, predictive validity could be achieved by models capturing the transition between high-risk, prodromal, and full expression of BD episodes.

#### Predictive validity

The degree to which manipulations of the model predict the effects of real-world interventions on the target condition of interest.

- There are identifiable and meaningful transitions between conditions/states of interest in the real-world phenomenon.
- Interventions/transitions in the model explain or predict corresponding transitions in the condition/state of interest.

The third and final subscale, concerning construct validity, evaluates the degree to which proposed models are constrained by empirically-derived biological or psychological architectures (Fig. 1). For instance, a simple autoregressive model or a complex recurrent neural network can be used to capture mood dynamics, as they are sufficiently general to explain most types of time-series data. However, that generality may not provide much causal insight into the biological or psychological mechanisms underlying the mood fluctuations in BD, unless specific components of these models are empirically or theoretically tied to biological or psychological constructs. In other words, a model with construct validity must have an architecture corresponding to biological or psychological properties found to be relevant in BD. For example, biophysical network models may allow us to understand the effects of neuronal excitability on circuit-level computations [24]. The results from these computational simulations can then be validated by examining results from real-world experiments.

#### Construct validity

The degree of homology between the model architecture and mechanisms that are empirically or theoretically deemed to underlie features of the target condition of interest.

- There is a real and identifiable or plausible mechanism underlying the target condition/state.
- The model architecture is homologous to the mechanism of interest, at an appropriate level of abstraction.

A model need not satisfy all three criteria to be useful. Face and predictive validity alone may confer clinical utility, with construct validity being irrelevant for such a model’s intended use. In fact, face validity alone may be of great benefit for characterizing features of BD.

### Application of the validity appraisal guide

Two authors (AN, SS) independently applied the VAG-CM to papers selected for full-text review. After an independent appraisal, the checklist responses for each paper were combined and inter-rater reliability was computed using Cohen’s *κ*. Our measurement and reporting of *κ* served to index the difficulty of interpreting each study in the context of their application to BD, and the likelihood with which we expect that alternative conclusions could be drawn by readers of respective papers. After inter-rater reliability analysis, we resolved conflicts by group consensus. We then applied hierarchical clustering in the R programming language (v. 4.1.2) to consensus checklist data to evaluate the consistency of ratings across similar studies, and to provide an organization for our narrative review.

## Results

A total of 128 titles were returned from the initial search and 20 additional papers were identified by searching reference lists. After title/abstract screening, 36 full-text papers remained, of which 26 met inclusion criteria. The ten excluded papers did not propose generative computational models of BD.

**Fig. 2:**
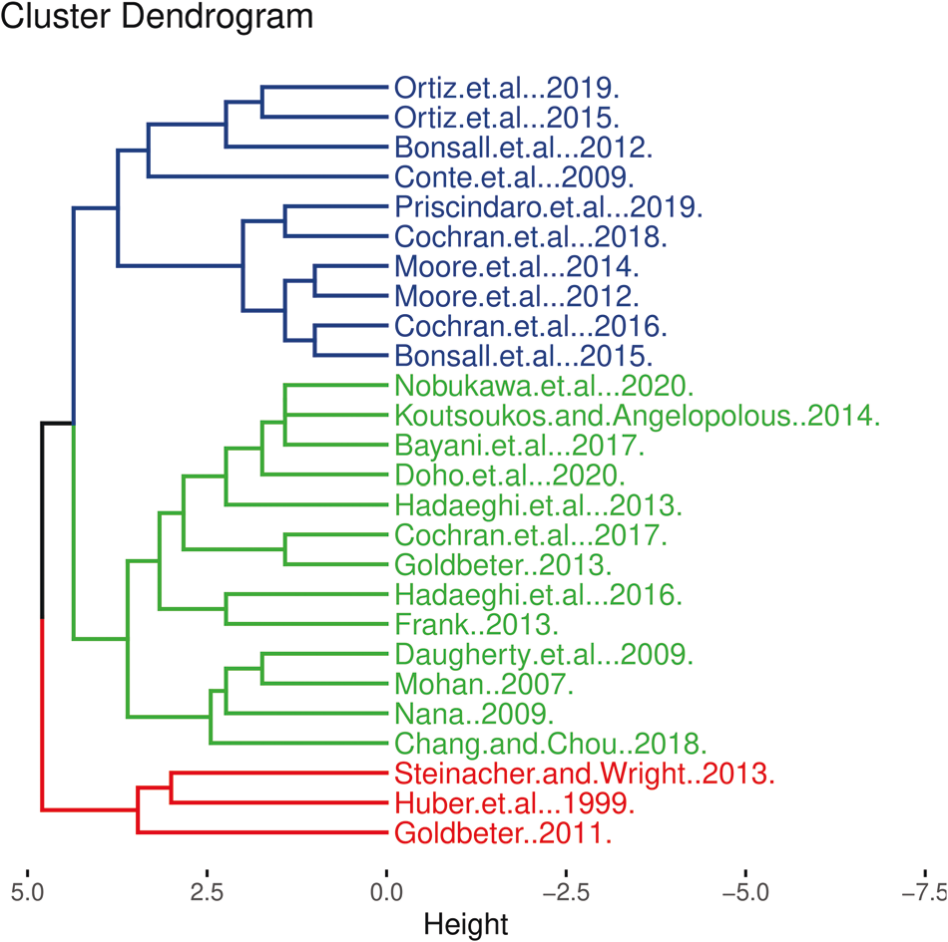
Dendrogram-based depiction of paper clustering according to results on the validity appraisal guide. Cluster 1 corresponds to largely data-driven models that showed strong face validity. Cluster 2 corresponds to studies presenting theory-driven models with predictive validity. Cluster 3 corresponds to studies presenting theory-driven models that largely lacked face, predictive, and construct validity.

An initial independent review of papers yielded a mean Cohen’s *κ* of 0.55, with a 95% confidence interval (CI) of (0.45, 0.64). Cohen’s *κ* values for individual papers are plotted in Fig. 2. A summary of VAG-CM ratings is shown in Table 1, with full results shown in Supplemental Table 1. However, we must emphasize that the VAG-CM is intended to be a guide for the structured appraisal of computational modeling studies and not a generalizable measure of absolute model validity. The level of inter-rater disagreement merely indicates the degree to which a given study was interpreted differently by the two raters.

**Table 1:** Summary of validity appraisal checklist results obtained by the two raters after resolution of discrepancies by consensus.

	Studies																									
	A	B	C	D	E	F	G	H	I	J	K	L	M	N	O	P	Q	R	S	T	U	V	W	X	Y	Z
Face validity																										
The model aims to describe a real-world phenomenon (i.e., a target state/condition vis a vis comparators)	1	1	1	1	1	0.5	1	1	0.5	1	1	0.5	0.5	1	0.5	1	1	0.5	1	0.5	1	0.5	1	1	0.5	0.5
The target condition being modeled is identifiable according to observable features	0.6	0.2	0.8	0.2	0.2	0.6	0.8	0	0.4	0	1	0	0.8	1	0.8	0.2	0	0.2	0.2	1	1	0.8	0.2	0	1	1
The comparator condition being modeled is identifiable according to observable features	0.7	0.2	0.7	0.3	0	0	0.8	0	0	0	1	0	0	1	0	0.2	0	0	0.3	0	1	0	0.2	0	0	0
The model actually explains or predicts the target condition vis a vis the comparator	0.1	0.1	0.4	0.1	0.1	0.1	0.4	0.1	0.3	0.1	0.4	0.1	0.4	0.1	0.4	0.1	0.1	0.1	0.1	0.7	0.4	0.7	0.1	0.1	0.4	0.4
Predictive validity																										
There are identifiable and meaningful transitions between conditions or states of interest in the real-world phenomenon	1	0.6	0.6	0.6	0.6	1	0	0	0	0.4	1	0	0	0	0	0	0	0	0.6	0	0	0	0	0	0	0
Interventions or transitions in the model explain or predict corresponding transitions in the condition of interest	0.6	0.4	0	0.4	0.8	0.8	0	0.4	0	0.2	0.8	0	0	0	0	0	0	0	0.4	0	0	0	0	0	0	0
Construct validity																										
There is a real and identifiable or plausible mechanism underlying the target condition	1	0	0	0	0	0	0	0.5	0	0	0	0	0	0	0	0.5	0	0	1	0	0	0	0	0.5	0	0
The model architecture is homologous to the mechanism of interest, at an appropriate level of abstraction	0	0.3	0.3	0.3	0	0.3	0	0.7	0.3	0	0	0	0.3	0.3	0	0.3	0.3	0.3	0	0.3	0	0	0	0	0.3	0

Results are split into sections concerning face validity, predictive validity, and construct validity. Each row indicates a specific subscale for the validity type being scored. Values depicted here are the proportions of affirmative responses across individual items within each subscale (the possible range is 0 to 1). Full results, including specific subscale items are shown in Supplemental Table 1. Legend*:* [A] Huber, Braun, and Krieg 1999; [B] Mohan 2007; [C] Conte et al. 2009; [D] Daugherty et al. 2009; [E] Nana 2009; [F] Goldbeter 2011; [G] Bonsall et al. 2012; [H] Frank 2013; [I] Goldbeter 2013; [J] Hadaeghi, Hashemi Golpayegani, and Gharibzadeh 2013; [K] Steinacher and Wright 2013; [L] Koutsoukos and Angelopoulos 2014; [M] Bonsall et al. 2015; [N] Ortiz et al. 2015; [O] Cochran, McInnis, and Forger 2016; [P] Hadaeghi et al. 2016; [Q] Bayani et al. 2017; [R] Cochran et al. 2017; [S] Chang and Chou 2018; [T] Cochran et al. 2018; [U] Ortiz et al. 2019; [V] Prisciandaro, Tolliver, and DeSantis 2019; [W] Doho et al. 2020; [X] Nobukawa et al. 2020; [Y] Moore et al. 2012; [Z] Moore et al. 2014.

Hierarchical clustering of VAG-CM data yielded three major groups of studies (Fig. 2) summarized by the following attributes: theory-driven models with predictive validity, theory-driven models lacking face, predictive, and construct validity, and data-driven models with face validity. These clusters demonstrate that studies employing similar approaches tend to be closely co-located on the dendrogram, indicating that a given study design or model resulted in consistent evaluations on the VAG-CM. For instance, Ortiz et al. [26, 27] both employed autoregressive (AR) models, as did Bonsall et al. [28] and Moore et al. [29, 30]. Bayani et al. [31], Nobukawa et al. [32], and Doho et al. [33] employed the same model architecture, as did several other closely co-located studies [34–37]. Three notable exceptions formed their own cluster of studies, owing to their demonstration of some degree of predictive validity [10, 21, 38]. Papers are summarized in Table 2.

**Table 2:** Description of models and corresponding data for verification.

Study	DD or TD	What it Models	Model type or method used	Data used to test model
Huber et al. [21]	TD	Episodes (binary occurrence vs. absence)	Deterministic and stochastic dynamical systems	None
Mohan [35]	TD	Mood oscillations/variations (untreated vs. treated)	Deterministic dynamical system	None
Conte et al. [11]	TD	Mood oscillations/variations (“latent” and “acclaimed phases”) Deterministic/stochastic contributions to mood variations in BD vs. healthy control.	Deterministic and stochastic dynamical systems	Qualitative description of the results of Gottschalk et al. [47]
Daugherty et al. [37]	TD	Mood oscillations/variations (treated vs. untreated, interactions between two patients with BD)	Deterministic dynamical system	None
Nana [34]	TD	Mood oscillations/variations (treated vs. untreated)	Deterministic dynamical system	None
Goldbeter [38]	TD	Mania and depression as independent, interacting systems. Mood oscillations/transitions (effect of antidepressants simulated)	Deterministic dynamical system	None
Bonsall et al. [28]	DD	Time-series of mood variability in stable and unstable BD (by fitting linear and nonlinear AR models to data)	Fitting linear and nonlinear AR models to data	QIDS-SR time-series (one measure per week over a 220-week period from 23 individuals with BD, divided into “stable mood” (*n* = 11) and “unstable mood” (*n* = 12)
Frank [41]	TD	Oscillations in second messenger systems	Deterministic dynamical system	None
Goldbeter [42]	TD	Mania and depression as independent, interacting systems. Mood oscillations/transitions (effect of antidepressants simulated)	Deterministic and stochastic dynamical systems	None
Hadaeghi, et al. [36]	TD	Mood oscillations/variations (treated vs. untreated)	Deterministic dynamical system	None
Steinacher & Wright [10]	TD	Time-course of behavioral activation/approach in BD, using both deterministic and stochastic systems	Deterministic and stochastic dynamical systems	Qualitative description of results from Wright et al. [50]
Koutsoukos & Angelopolous [51]	TD	Energy (mood) oscillations/variations generated from a theoretical mood “pendulum” (effect of mood-stabilizers considered)	Deterministic dynamical system	None
Bonsall et al. [39]	DD + TD	Time-series of mood variability (by fitting linear and threshold AR models to time-series data). Mood fluctuations using both deterministic and stochastic dynamical systems (relaxation oscillators fit to time-series data)	Fitting linear and threshold AR models to data Deterministic and stochastic dynamical systems	QIDS-SR time-series from 61 individuals with BD (one measure per week for 79–233 weeks). *n* = 42 used for AR models, *n* = 19 for relaxation oscillator models.
Ortiz et al. [26]	DD	Time-series of mood, anxiety and energy in BD vs. healthy control (by fitting AR models to time-series data).	Fitting AR models to time-series data	Time-series data of self-reported mood, anxiety and energy levels using a visual analog scale from 30 individuals with BD, and 30 healthy controls, (two measures per day, for 90 days)
Cochran et al. [44]	DD	Clinical course of BD by fitting discrete-time Markov chain model with discretized mood states to longitudinal data.	Discrete-time Markov chain model	Data from the Prechter Longitudinal Study of Bipolar Disorder at the University of Michigan [58] (*n* = 209 individuals with bipolar I disorder)
Hadaeghi et al. [52]	TD	Circadian activity variation in BD	Deterministic dynamical system	Actigraphic data from n=15 subjects, but model not fit to group level data, and comparisons between model output and data are shown for single subject only.
Bayani et al. [31]	TD	Circadian activity pulse trains in BD	Deterministic dynamical system	None
Cochran et al. [40]	DD + TD	Mood variations	Patient-level statistics to test a set of hypotheses, followed by a proposed stochastic dynamical system	Self-report ASRM and Patient Health Questionnaire for Depression (PHQ-9), collected every 2 months from 178 individuals with BD, for at least 4 years
Chang & Chou [53]	TD	Relationship between mood sensitivity and realized/expected value. Simulated QIDS-SR16 scores.	Deterministic dynamical system	None
Cochran et al. [78]	TD	Time-course of mood variations in BD using stochastic models	Stochastic dynamical systems	None
Ortiz et al. [27]	DD	Time-series of mood, anxiety and energy in BD, unaffected first-degree relatives, and healthy controls (by fitting AR models to time-series data).	Fitting AR models to time-series data	Time-series data of self-reported mood, anxiety and energy levels using a visual analog scale (two measures per day, for 90 days) in 30 individuals with BD, 30 unaffected first-degree relatives and 30 healthy controls
Prisciandaro et al. [43]	DD	Empirically-derived mood states and transition probabilities in BD (using hidden Markov modeling)	Hidden Markov modeling	Longitudinal data from STEP-BD study [79] (*n* = 3918 for transition probability analyses, n=3229 for analyses involving baseline covariates)
Doho et al. [33]	TD	Neural activity related to circadian function in BD and the effect of chronotherapy on neuronal activity	Deterministic dynamical system	None
Nobukawa et al. [32]	TD	Frontal neural activity and circadian activity in BD and healthy control, and effect of chronotherapy	Deterministic and stochastic dynamical systems	None
Moore et al. [30]	DD	Forecasting time-series of QIDS-SR scores in BD	Fitting statistical models to time-series data. Forecasting using AR, exponential smoothing, Gaussian process regression	QIDS-SR and ASRM time-series from 100 individuals with BD (one measure per week for 3.5 years). Only QIDS-SR scores were used for forecasting.
Moore et al. [29]	DD	Forecasting time-series of QIDS-SR scores in BD	Fitting statistical models to time-series data. Linear and nonlinear forecasting using: persistence, exponential smoothing, AR, gaussian process regression, locally constant prediction, local linear prediction	QIDS-SR time-series from eight individuals with BD (one measure per week for 5 years)

*AR* autoregressive, *ASRM* Altman self-rating mania scale, *BD* bipolar disorder, *DD* data-driven, *PHQ-9* patient health questionnaire, *QIDS-SR* quick inventory of depressive symptoms, *STEP-BD* systematic treatment enhancement program for bipolar disorder, *TD* theory-driven.

### Primarily data-driven models with face validity

Many studies employed AR models which assume that the value of mood (or energy, activation, etc.) at time *t* is a linear function of its own values at the previous 1 ≤ *k* ≤ *t* recordings. Although not constructed to be explicitly homologous with any physiological or psychological systems, AR models offer statistically rigorous explanations of time-series data in BD [26–30, 39]. Most of these models showed that self-ratings from BD are often well predicted by self-ratings at the previous time step (i.e., the AR[1] model) [30, 39] (Cohen’s *κ* = 0.8 and 0.54, respectively), although this is likely also the case for healthy controls [26] (*κ* = 0.94) and unaffected first-degree relatives of BD patients [27] (*κ* = 0.88).

Other studies that demonstrated face validity used models based on coupled stochastic differential equations, which do not assume that mood is linearly dependent on its past values. Using self-report data collected every 2 months from 178 BD patients, Cochran et al. [40]. (*κ* = 0.42) evaluated several predictions made by existing theoretical models of mood dynamics in BD. The first assumption they tested was that mood fluctuations are inherently rhythmic with a consistent period [34, 37–39, 41, 42], which their data could not conclusively prove or disprove. Although they found no consistently predominant mood oscillation frequency across subjects, it is possible that the dominant frequency of mood oscillations varies across subjects. Their study was insufficiently powered to detect such differences after controlling for multiple comparisons.

The second assumption tested by Cochran et al. [40]. was that mood states in BD such as mania, depression, and euthymia are themselves stable states (attractors) in which patients’ moods become stuck, generating multiple modes in the distribution of mood ratings [10, 38, 42]. Here, their data supported a unimodal hypothesis, whereby mood episodes are likely best captured as extremes of mood fluctuation.

The third assumption tested by Cochran et al. [40]. was that mood is a one-dimensional construct with depression and mania at opposite poles [10, 28, 34, 37, 39]. This hypothesis was inconsistent with their data, given the occurrence of mixed episodes. These findings led them to develop the affective instability model, which assumes that mania and depression are governed separately, but may be positively or negatively correlated. Their model may thus account for mixed episodes (positive correlation between mania and depression) or shifts between exclusively manic or depressed states (negative correlation between mania and depression). Their model assumes that BD does not have intrinsic periodicity. Rather, they predict that individuals can end up in pathological mood states for two reasons: (A) a patient’s baseline mood is already close to pathological levels, making a patient vulnerable to small mood perturbations, or (B) mood is particularly sensitive and reaches pathological levels easily.

Markov Chain models [43, 44] with finite states (either observable or latent) identified discrete episodes (euthymia, depression, and mixed states) in longitudinal continuous self-report data [43], and that the pattern of mood state transition probabilities across subjects may relate to suicide attempts, disability, and disease chronicity [44].

One study used a van der Pol oscillator [45, 46] model to directly explain the findings of Gottschalk, Bauer, and Whybrow [47] that mood dynamics in BD are governed by a system that is more deterministic (less random) than that observed in healthy controls [11] (*κ* = 0.38). This model suggests that biological systems governing mood in BD either lack internal randomness, or are resistant to external sources of noise.

One study hypothesized that mood trajectories in BD are generated by two separate oscillating systems that can be coupled with varying degrees of strength [39] (*κ* = 0.54). This model was fit to QIDS-SR data collected from 25 patients with BD, ultimately predicting that if a system of neural oscillators underlying mood dynamics can be identified, they will oscillate independently of each other in the majority of BD patients. This prediction may be supported by existing empirically-derived models of hemispheric mood lateralization [48, 49].

### Theory-driven models with predictive validity

The studies discussed in this section are primarily theory-driven models with predictive validity, meaning the features chosen to be modeled were operationalized and empirically identifiable. A modified single neuron model was used to describe mood episodes as binary events (see Supplemental Materials for details), capturing phases of BD progression and the kindling phenomenon, whereby mood episodes become progressively autonomous and frequent (*κ* = 0.35) [21]. Illness progression was modeled using a scalar parameter *S* monotonically representing the illness stage. Early in the disease course (i.e., at low values of *S*), the model’s inter-episode interval was highly sensitive to exogenous noise, but gradually developed periodic and then chaotic episodicity, independent of external perturbations. These dynamics may predict that early in the BD disease course, individuals with more stressful life events (e.g., external perturbations) should show greater aperiodicity in episodes, compared to individuals with few stressful life events. As time progresses, the model predicts that most individuals will enter a stable periodic regime with relative insensitivity to life events, followed by multistable and chaotic regimes late in the illness.

Another study proposed a set of models assuming that mania and depression were independent attractor states, between which an individual’s mood could oscillate [38, 42] (*κ* = 0.65 and 0.43, respectively). This model proposed by Goldbeter [38] predicted that if a depressed patient were given a sufficiently high antidepressant dose, the system governing mood oscillations would enter the bistable region, in which a manic switch could occur. The Goldbeter [38, 42] model makes a prediction that could be experimentally scrutinized: that an individual’s risk of antidepressant-induced manic switch in bipolar depression should be dose-dependent.

Only one model in this review captured inter-episodic euthymia along with mania and depression [10] (*κ* = 0.68). Steinacher and Wright [10] modeled behavioral activation as a scalar value *X* whose change over time is proportional to *X*^n^, where *n* ≥ 0 is a parameter governing “self-excitation” of the behavioral activation system (BAS). When *n* = 1, the contribution of *X* to self-excitation is linear. When *n* > 1, a phenomenon of synergistic activation will be observed, where each additional unit increase of *X* will disproportionately increase the amount of self-excitation (e.g., activation begets more activation). Finally, when *n* < 1, each unit of increase in *X* produces marginally less self-excitation.

The Steinacher and Wright [10] model predicts that hypersensitivity of the BAS, indexed by *n*, may relate to the propensity for mood episodes [12]. The authors compared BAS recovery time in their model to published data in which BAS recovery time is prolonged in BD patients with more mood episodes [50]. Thus, Steinacher and Wright [10] implicitly predict that their models, if fit to longitudinal BAS recordings from BD patients, should recover values of *n* in proportion to patients’ respective number of mood episodes.

The studies described in this section shared the common strength of building models to explain features of BD that were cited from relevant literature. However, a common limitation is that no model was designed with an architecture based on biological or psychological substrates of BD, thus limiting construct validity.

### Theory-driven models lacking face, predictive, and construct validity

Several models attempted to capture mood oscillations [34–37, 41, 51] or circadian variations of activity in BD [31–33, 52]. We will not review each study in detail, but rather discuss common features that limit the achievement of face, predictive, or construct validity. Most prominently, most studies failed to clearly define features that would be representative of the target condition (usually BD) or comparators being modeled. Often, “mood oscillations” were presented as the sine qua non of BD, without clear operationalization or differentiation from patterns of mood fluctuation in other conditions or healthy individuals. That is, many papers simply referred to “mood” without operationalizing what “mood” is or why it fluctuates. Most importantly, such studies did not provide empirical or theoretical justification for why certain patterns of mood fluctuation are characteristic of BD vs. other conditions.

The study by Chang and Chou [53] (*κ* = 0.38) may be an exception to the other papers in this cluster. Their model was an extension of existing reinforcement learning-based dynamical systems models, which propose that mood variation relates to discrepancies between the amount of value an individual receives vs. how much is expected [13–16]. Using reinforcement learning as the conceptual thrust behind their model architecture offers some potential construct validity, since such models can be compared to behavioral and physiological recordings simultaneously [14, 16]. Ultimately this study was rated low on the VAG-CM construct validity subscale because aspects of their model were justified based on mathematical convenience without motivation by, or prediction of, theoretical or empirical observations related to BD. Furthermore, they provided little justification for the assumption that “mood oscillation” is the defining feature of BD, which by itself is not a well-defined concept.

That being said, let us, for the moment, accept the definition of BD as the presence of self-sustained mood oscillations. Chang and Chou [53] argued that such a situation would arise from hypersensitivity to random external perturbations (noise) insofar as this noise generates prediction errors (differences between expectation and reality). They predict that self-sustained mood oscillations are induced when mood sensitivity exceeds a critical threshold, after which mood is excessively sensitive to random noise in the environment. This model implicitly predicts that, in patients with presumed unipolar depression, the rate of antidepressant-induced manic switch should be proportional to the increase in mood sensitivity experienced during the antidepressant treatment.

## Discussion

We have presented a critical review of dynamical systems models of BD using a novel validity appraisal guide for computational models inspired by the animal model literature [22]. While studies did not yield a common unified perspective on the temporal course of BD, a methodological theme emerged. Specifically, our approach led us to identify a need to combine data-driven and theory-driven approaches during the development of dynamical systems models of BD. Future studies should (A) use data-driven approaches to identify critical features of BD and relevant comparators and (B) develop generative models to explain those features. Preferably, the latter generative models should be developed homologously to physiological or psychological systems involved in BD. This need to align models with experiment further highlights the need for longitudinal studies of BD using multimodal data collection such as passive sensing, mood ratings, physiological signals (e.g., heart rate and variability, galvanic skin response), or ecological momentary assessment methods [54]. In the remainder of this discussion, we present a roadmap for this line of research.

Dynamical systems modeling studies in BD must clearly identify the condition and comparators of interest and the features that define them. That is, we must rigorously attempt to define some notion of a “ground truth” concerning the nature of BD (at least with respect to a subset of features; Fig. 1, Panels B1-2 and C1-2). Accomplishing this will involve collecting relevant, high-quality data from well-characterized and matched subject groups, such as BD probands, unaffected relatives, and/or controls, as was done by Ortiz et al. [27]. Unfortunately, many studies did not include comparator states, which precludes any assessment of their discriminant, and consequently face, validity. Future studies that collect longitudinal time-series data from well-characterized BD samples should also aim to capture relevant phases of the illness, including the early high-risk period [55], illness onset [56, 57], and long-term maintenance in naturalistic settings [58].

Features deemed central to the explanation of target and comparator conditions must also be defined a priori. The identification of multiple features would be ideal since this contributes further to the convergent validity of an explanatory model. Studies employing AR models were generally excellent in this respect, using multiple descriptive statistics to identify features of BD time-series. For example, Ortiz et al. [26]. showed that mood, anxiety, and energy self-ratings during euthymia are Gaussian distributed, and that mood and anxiety are negatively cross-correlated (increases in mood are associated with reduction of anxiety). Using similar approaches, Ortiz et al. [27]. found lower multiscale entropy levels in mood and energy time-series from euthymic BD patients and unaffected first-degree relatives, compared to healthy controls. Interestingly, time-series entropy in mood self-reports has been shown to increase in the 60 days prior to a manic or depressive episode, compared to the 60 days prior to a month of euthymia [59]. Future computational modeling research should attempt to build mechanistic generative models of mood dynamics that can explain these features. Thus, longitudinal studies must collect sufficient data to capture multiple aspects of BD, such as variations in activity, cognition, emotion, and physiology [54]. Recordings must also cover manic, depressive, and euthymic phases, as well as longer-term illness progression. Designing and funding such studies is a pressing challenge [60–63].

After defining target and comparator conditions, along with central features to be explained, future studies must ensure that proposed models are appropriately fit to these data. The optimal approach would be to fit the generative models to raw data, as was done by several previous authors [26–30, 39, 40, 43, 44]. Alternatively, one may consider estimating the degree to which generative models can produce behavior replicating the results of data-driven studies in this review. We did not employ this approach in the present study since our primary focus was broader, and included an evaluation of biological or psychological homology (construct validity), which is not always based on statistical considerations. Furthermore, we do not believe that statistically fitting a model to raw or summary data is always necessary. Depending on the question, it may suffice to show that a model generates behavior corresponding qualitatively to empirical observations [64]. For instance, Steinacher and Wright [10] showed that increasing the BAS self-activation parameter *n* resulted in prolonged behavioral activation recovery times after perturbation of their model, similar to that observed in BD patients with many prior episodes. Although not fit statistically to raw data, their model explained a clear effect from cited literature, which is nonetheless useful.

The discussion up until now proposes an inductive approach to model building, whereby data are collected first, and a model is built to explain those data. We also believe that a deductive approach is worthwhile, where a model is built first and its predictions are subsequently tested through experimentation. Indeed, in this paper, we recommend empirical testing of predictions made by models from Chang & Chou [53] and Huber et al. [21]. However, when modeling first, and testing predictions second, we must remember that the assumptions underpinning the model must be empirically supported. It may be tempting to axiomatically presume that “mood oscillations define BD,” and subsequently introduce any number of oscillating systems [34–37, 41, 51] whose components could very well be labeled with the names of brain areas [31–33, 52], despite no justifiable architectural homology (i.e. no construct validity). Rather, we must ensure that assumptions underlying theoretical models are derived from known clinical characteristics (qualitative or quantitative) about the natural system the model seeks to explain. We believe this was best outlined by the late Robert Rosen in his seminal text Anticipatory Systems [65].

*“[A model] is a relation between a natural system and a formal system. This relation [is] established through an appropriate encoding of the qualities of the natural system into the propositions of the formal system, in such a way that the inferential structure of the latter correspond to the system laws of the former.”*

In other words, if a model’s assumptions are not grounded in reliable and relevant facts about BD, then the model’s ability to capture the system laws underpinning BD is doubtful. Consequently, we emphasize the importance of close collaboration between computational researchers and clinical and basic scientists, who ultimately pursue and provide necessary observations of the natural system of BD.

To this end, face validity need not be the first form of validity established. A worthwhile and much-needed alternative approach is to first build a model with construct validity: that is, to build a model whose architecture captures the structure or function of relevant biological or psychological systems in BD. For instance, it would be interesting and useful to build a biologically detailed model of the circadian system that includes exogenous inputs simulating blue light [66] and objects representing key molecules involved in both circadian rhythms and response to mood-stabilizers [67, 68]. Such a biologically realistic model would have construct validity based on its design being homologous to the natural circadian system of interest. If a modeler were to clearly articulate the importance of chronobiological mechanisms in BD through citation or direct experimental observation [67], then the predictions made by such a model would be worthwhile to verify empirically.

That being said, establishing the homology and construct validity of a computational model is insufficient to ascribe value to its predictions. Those predictions are only valuable if they are specific, operationalized, and ultimately testable. In other words, modelers must specify how their simulation’s predicted behaviors translate into defined observations of the natural system of interest (here BD). For instance, even though second messenger signaling systems are involved in BD [69–71], simply modeling the behavior of these systems will tell us little about BD [41]. The relevance of such a model’s behavior for BD requires a clear understanding of how those behaviors translate into observable phenomena in BD. Thus, if a model makes predictions about BD that are meant to be tested empirically, then the modeler must articulate those predictions in terms of what should be observed in BD patients.

Finally, dynamical systems models of BD should strive to achieve predictive validity. As an example, one may consider modeling the results of Glenn et al.[59], who consider the change in affective dynamics occurring in the lead-up to mood episodes. One possible candidate for such a model is that of Chang and Chou [53], which predicts that the increased entropy in the 60 days preceding a mood episode would be related to heightened mood sensitivity. Since the architecture and dynamics of the Chang and Chou [53] model are theoretically and empirically motivated by reinforcement learning, one could plausibly test this hypothesis in the laboratory by fitting the model to behavioral and physiological signals simultaneously, thereby enhancing both face and construct validity [14, 16, 72–74].

A strength of the present review was the guided evaluation of studies by multiple raters using an a priori-defined checklist based on accepted criteria in the animal modeling literature [22]. We must reiterate that this checklist is not intended, nor should it be treated, as a validated measure of the absolute quality of a computational model. Rather, it is intended to provide a structured approach to the appraisal of computational psychiatric models. The papers included in our review showed remarkable heterogeneity in their methodology and reporting. Some papers were clearly written primarily for applied mathematical audiences, while others were geared toward clinical readerships. Without a structured approach to appraising these studies, one could speculatively attribute clinical relevance to mathematical papers that claimed to model BD, but instead presented technical results about general oscillatory dynamics [34–37, 41, 51]. Conversely, for studies that focus on showing strong statistical descriptions of time-series data from real patients, one may easily overlook the lack of biological or psychological homology in the corresponding dynamical systems model [26–30, 39]. Our VAG-CM was therefore intended to ensure that (A) reviewers agreed a priori on what was important to evaluate in a modeling study, and (B) disagreements could be easily identified and efficiently discussed. Although we applied the VAG-CM specifically to dynamical systems models of BD, it would be of interest to evaluate its utility more broadly for model appraisal in computational psychiatry. Furthermore, we encourage developments to the VAG-CM that are practically applicable for experimental and computational modeling researchers, and that consider alternative perspectives on model validity, such as those from philosophy of neuroscience [75, 76].

One limitation of our review is the inclusion of studies modeling different measures, collected at different durations and frequencies. This limited our ability to make direct comparisons of predictions across papers. Another limitation of our review may be that some included papers were evaluated with low inter-rater reliability after initial screening. However, the poor inter-rater reliability obtained for some studies may simply reflect that the aims, design, and outcomes were reported in such a fashion that caused reviewers to draw different conclusions. Consequently, we believe that those studies with the low inter-rater agreement may also be interpreted differently by readers. It is also possible that the VAG-CM is a poor appraisal tool in a general sense. However, since there are currently no viable alternative methods for the structured appraisal of the diverse computational modeling approaches in psychiatry, we believe our VAG-CM is a pragmatic and conservative approach. The development of reporting and appraisal standards for computational modeling studies in psychiatry is a priority in order to maximize the quality and impact of this promising research area. Thus, we encourage future studies to evaluate, extend, or revise the VAG-CM, with detailed and extensive studies of its inter-rater reliability and generalizability to other disorders and model types (perhaps using a database of computational modeling studies such as CPSYMAP [77]). Such an evaluation was beyond the scope of the present study, as was a larger study of inter-rater reliability, since the VAG-CM here served solely as a tool to approach our review.

In conclusion, by approaching our review of dynamical systems models of BD in a structured fashion (using the VAG-CM), we have identified a disconnect between the data-driven and theory-driven approaches used in the BD modeling literature. We argue that taking a blended approach that combines the strengths of both data-driven and theory-driven methods will ensure future models adequately explain behavior in BD, generate results that can be empirically verified, and provide mechanistic insights into BD through homology with biological and psychological systems. By developing biologically or psychologically homologous models of the BD phenotype, we will step closer toward understanding how the brain generates this severe condition, and how its management can be improved.

## Supplementary information

Supplemental Materials
