# Supplementary material for: A critical evaluation of dynamical systems models of bipolar disorder
Source: Transl Psychiatry. 2022 Sep 28;12:416. doi: 10.1038/s41398-022-02194-4 (PMC9519533; doi:10.1038/s41398-022-02194-4)
Supplement: Supplementary file 1 — Supplemental Materials [file 41398_2022_2194_MOESM1_ESM.pdf]

Supplemental Materials for *A Critical Evaluation of Dynamical Systems Models of Bipolar Disorder*

Abraham Nunes[1,2,*], Selena Singh[3], Jared Allman[1], Suzanna Becker[3], Abigail Ortiz[4,5], Thomas Trappenberg[2], and Martin Alda[1]

[1] *Department of Psychiatry, Dalhousie University, Halifax, Nova Scotia, Canada*
[2] *Faculty of Computer Science, Dalhousie University, Halifax, Nova Scotia, Canada*
[3] *Department of Psychology, Neuroscience & Behaviour, McMaster University, Hamilton, Ontario, Canada*
[4] *Department of Psychiatry, University of Toronto, Toronto, ON, Canada.*
[5] *Centre for Addiction & Mental Health, Toronto, Ontario, Canada*
[*] *Correspondence: Abraham Nunes (nunes@dal.ca). 3083B-5909 Veterans Memorial Lane, Abbie J. Lane Memorial Building, QEII Health Sciences Centre, Halifax, Nova Scotia, B3H 2E2, Canada*

CONTENTS

1 Overview of Dynamical Systems **2**

2 Validity Appraisal Guide for Computational Models **6**
- 2.1 Subscale 1: Face Validity 6
- 2.2 Subscale 2: Predictive Validity 6
- 2.3 Subscale 3: Construct Validity 7

3 Inter-Rater Reliability Results **8**

4 Validity Appraisal Guide Results Table **8**

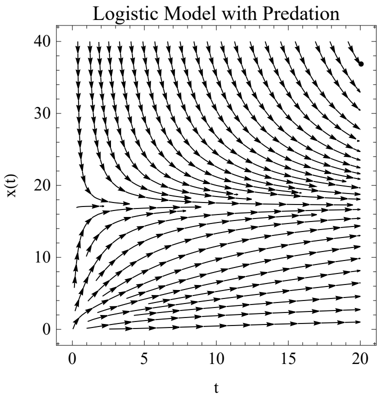

Supplemental Figure 1: Illustration of the different time-courses of growth of a population subject to predation, as expressed by the differential equation $\dot{x} = ax(1 - x/x_{\max}) - bx^2/(c^2 + x^2)$, with parameters $a = 2, x_{\max} = 20, b = 5, c = 2$. Each curve represents the trajectory of a population given initial value $x(0) = x_0$. Arrows denote the direction of time.

1 OVERVIEW OF DYNAMICAL SYSTEMS

In order to facilitate interpretation of the models uncovered by our review, this section first introduces some basic technical aspects of dynamical systems using simple examples. A dynamical system is a system whose state evolves over time in accordance to an "evolution rule", which can be mathematically described by sets of *differential equations* in the continuous setting, or *difference equations* in the discrete setting. Here, we provide a basic introduction to models based on differential equations.

Our first model is demonstrative of continuous dynamical systems that reach a stable equilibrium (a *stable state* or *fixed point*). These models may have one or more stable states, but are characterized by the system converging to, and remaining at, these points or limit sets until perturbed. The specific stable state to which a system arrives may depend on several factors, including the starting point. Example 1 describes a simple dynamical system modelling growth of a population under (A) space constraints and (B) predation, in which the population size reaches a stable equilibrium.

Example 1 (A System with Stable States)**.** *A classical dynamical system describes the growth of a population whose size at time* $0 \leq t$ *is denoted* $x(t)$ *or simply* x *for parsimony. The rate of change in* x*, denoted* $\dot{x}$ *(alternatively* $x'(t)$ *or* $\mathrm{d}x/\mathrm{d}t$*) is*

$$\dot{x} = a\left(1 - \frac{x}{x_{\max}}\right)x, \tag{1}$$

which is known as the logistic equation. Here, coefficient $x_{\max}$ *denotes the maximum possible population size, and* a *is a scalar value identifying the growth rate. If* $a > 1$*, then the population will grow over time, whereas it will shrink if* $a < 1$*. The term* $(1 - x/x_{\max})$ *indicates the proportion of population capacity still available for reproduction. As* x *approaches* $x_{\max}$*, the value of* $(1 - x/x_{\max})$ *approaches 0 and reproduction stops.*

Some populations are also subject to predation. This can be incorporated into Equation 1 by adding a predation function $bx^2/(c^2 + x^2)$*, where* $b > 0$ *is a constant representing the rate of predation, and* $c > 0$ *represents the size of the population at which 50% of the maximum predation rate is reached (note that this is equivalent to the biochemical Hill function with exponent* $n = 2$*). Equation 1 thus becomes*

$$\dot{x} = a\left(1 - \frac{x}{x_{\max}}\right)x - b\frac{x^2}{c^2 + x^2}. \tag{2}$$

Supplemental Figure 1 is a stream plot showing the trajectories of population size (as modelled by Equation 2) across time. For initial population sizes greater than 0, the population will converge on a stable equilibrium value. The rate at which the population approaches that fixed point is dependent on the initial population size.

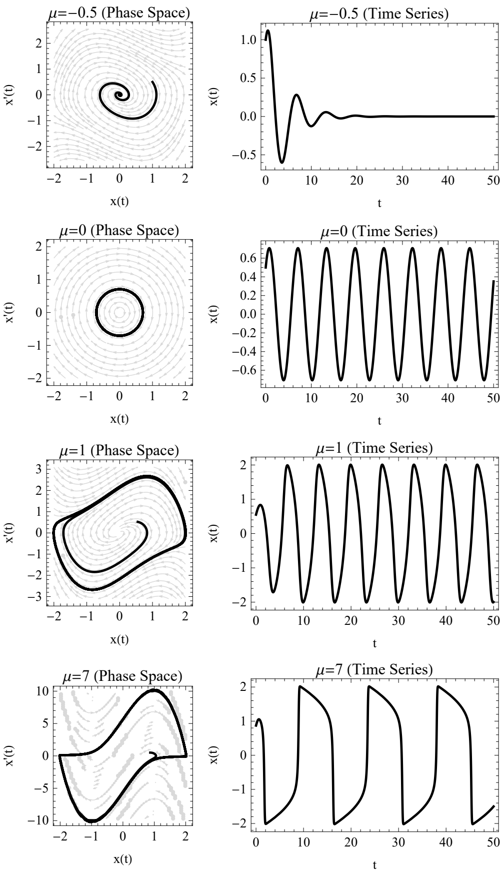

Supplemental Figure 2: The van der Pol oscillator (VPO) [8, 9] across values of the nonlinearity/damping parameter μ. Each row of plots illustrates a VPO for different values of μ. The left and right columns show the phase space portraits, and corresponding time series, respectively.

Instead of demonstrating stable fixed points, some systems show solutions that converge to limit cycle attractors, resulting in periodic oscillations. One such class of systems that will be encountered across many studies in our review [1–7] is the *relaxation oscillator* (RO), most notably the *van der Pol oscillator* (VPO) [8, 9]. Although population dynamics models, including those adapted for analysis of biochemical kinetics can also produce oscillatory behaviour [10, 11], Example 2 will introduce the VPO.

Example 2 (A System with Periodic Oscillations)**.** *A van der Pol oscillator [8, 9] is a one-dimensional system with state $x(t)$ (here simply x) is defined according to the following second order ordinary differential equation:*

$$\ddot{x} = \mu(1 - x^2)\dot{x} - x, \tag{3}$$

where "second order" refers to the fact that these dynamics are expressed in terms of the second derivative of x with respect to time: $\ddot{x} = x''(t) = \mathrm{d}^2x/\mathrm{d}t^2$. The parameter μ is a scalar value representing the amount of damping or nonlinearity. Supplemental Figure 2 shows the VPO behaviour across different values of μ. When $\mu < 0$, the system will show a progressive damping of oscillations until x approaches 0. When $\mu = 0$, the system shows simple harmonic motion. When $\mu > 0$, we observe limit cycles (stable periodic trajectories to which the system will converge from multiple different starting points).

Supplemental Figure 2 also demonstrates the phenomenon of a "relaxation oscillation," whereby the system alternates between slow and rapid movements (depicted here when $\mu = 7$).

One can appreciate why RO models might be proposed for BD mood dynamics. Superficially, BD consists of episodes in which an individual persists for some time, followed ostensibly by transition into a

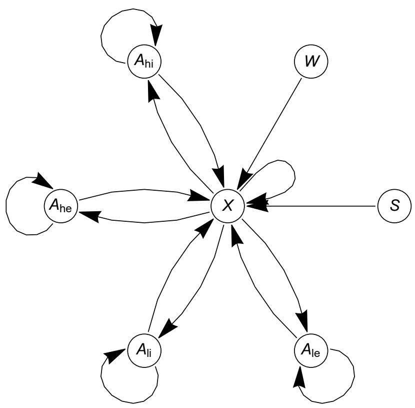

Supplemental Figure 3: Graphical illustration of the Huber *et al.* [1] model.

different state of relative stability. However, natural phenomena rarely proceed with such regular periodicity. Rather, naturally oscillating systems are often characterized by behaviour that appears almost random. Yet, seemingly random behaviour can emerge from a totally deterministic system in a phenomenon known as *chaotic dynamics*.

Example 3 (A Chaotic Continuous Dynamical System)**.** *Huber* et al. *[1] sought to model episodes of mania and depression as discrete (on/off) events, since their primary concern was the rhythmicity of episodes and inter-episode timing. This was implemented using a model of neuronal spiking illustrated graphically in Supplemental Figure 3, and characterized by the following ordinary differential equation,*

$$\tau_x \dot{x} = -x - \sum_{i \in \mathcal{A}} A_i^v w_i (x - x_i) + S + W \tag{4}$$

where x is analogous to a neuronal membrane potential, τ_x is a relaxation time constant, S is a constant external input, and W is Gaussian noise. The set $\mathcal{A} = \{\mathrm{he}, \mathrm{hi}, \mathrm{le}, \mathrm{li}\}$ identifies different excitatory and inhibitory elements of the system. That is, the system contains high-threshold excitatory and inhibitory elements A_{he} and A_{hi}, as well as low-threshold excitatory and inhibitory elements A_{le} and A_{li}, respectively. These are coupled to the state variable x according to "synaptic weights" $w_i \in \{w_{\mathrm{he}}, w_{\mathrm{hi}}, w_{\mathrm{le}}, w_{\mathrm{li}}\}$ and activation threshold constants $x_i \in \{x_{\mathrm{he}}, x_{\mathrm{hi}}, x_{\mathrm{le}}, x_{\mathrm{li}}\}$. Huber et al. *[1] set the exponent v to 2 for A_{he}, and to 1 otherwise.*

The system simulated by Huber et al. *[1] can be conceptualized as a mixture of excitatory/inhibitory oscillating subsystems, whose states $A_i \in \{A_{\mathrm{he}}, A_{\mathrm{hi}}, A_{\mathrm{le}}, A_{\mathrm{li}}\}$ themselves evolve over time according to the following differential equation:*

$$\tau_i \dot{A}_i = f_i(x) - A_i. \tag{5}$$

Here, τ_i is a relaxation time constant, and

$$f_i(x) = \frac{1}{1 + \exp\{\beta_i (x - x_{i,0.5})\}} \tag{6}$$

is a sigmoid with slope β_i and half-activation constant $x_{i,0.5}$.

At different levels of deterministic input (S), this system is capable of simulating fixed-point or steady state dynamics, as well as periodic oscillations, limit cycles, and chaotic behaviour. Supplemental Figure 4 demonstrates this behaviour. With $S \approx 10$ or below, the system undergoes dampened oscillations to a limiting fixed point. At $S \approx 50$, we observe regular periodic spiking, and periodic bursts at $S \approx 100$. At levels of S between approximately 175 and 325, we observe chaotic inter-spike intervals (see Huber et al. *[1] for further detail through bifurcation plots).*

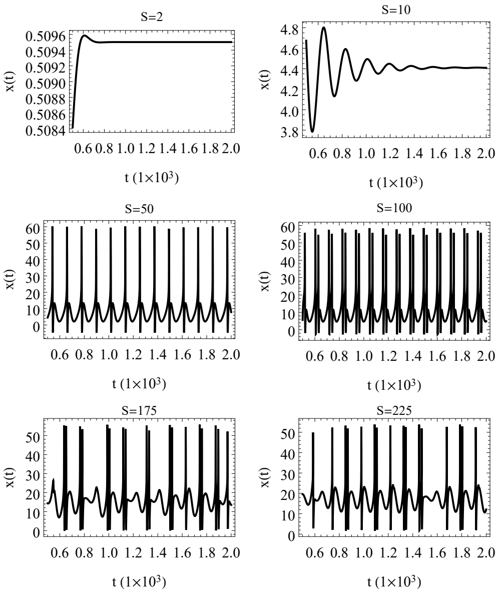

Supplemental Figure 4: Results of deterministc simulations of the Huber *et al.* [1] model at various levels of constant input S. This simulation was generated with the following parameters: $\tau_x = 10$, $\tau_{\mathrm{hi}} = 2$, $\tau_{\mathrm{li}} = 100$, $\tau_{\mathrm{le}} = 10$, $x_{\mathrm{hi}} = -30$, $x_{\mathrm{he}} = 110$, $x_{\mathrm{li}} = -30$, $x_{\mathrm{le}} = 110$, $w_{\mathrm{hi}} = 20$, $w_{\mathrm{he}} = 15$, $w_{\mathrm{li}} = 18$, $w_{\mathrm{le}} = 3$, and initial conditions $x(0) = A_i(0) = 0$ for all $i \in \{\mathrm{he}, \mathrm{hi}, \mathrm{le}, \mathrm{li}\}$.

In this section, we have introduced simple examples of continuous dynamical systems capable of generating multiple forms of behaviour, including fixed-points, stable oscillations and limit cycles, as well as chaotic dynamics. Two of these models, the RO (Example 2) and neuronal model (Example 3), are directly implemented in studies that will be discussed in the Results and Discussion of the main body.

2 VALIDITY APPRAISAL GUIDE FOR COMPUTATIONAL MODELS

2.1 Subscale 1: Face Validity

Definition: The degree to which the model exhibits a range of behaviours similar to that of the condition of interest In other words, how good is the model at explaining behaviour in the target condition?

1. The model aims to describe a real-world phenomenon (i.e. a target state/condition *vis a vis* comparators)

   ☐ Target condition defined clearly

   ☐ Comparator(s) defined clearly

2. The target state/condition being modelled is identifiable according to observable features

   ☐ Features are explicitly defined

   ☐ Feature definitions are operationalized with concrete criteria or measurement scales

   ☐ Relevance of features to condition of interest is stated (i.e. features actually present clinically)

   ☐ Features are identified based on cited empirical observations or experiments

   ☐ Multiple features considered

3. The comparator state/condition being modelled is identifiable according to observable features

   ☐ Features are explicitly defined

   ☐ Feature definitions are operationalized

   ☐ Relevance of features to condition of interest is stated (i.e. features actually present clinically)

   ☐ Features are identified based on cited empirical observations or experiments

   ☐ Multiple features considered

   ☐ Relationship to features of target condition identified (e.g. mutual exclusivity, correlation, etc.)

4. The model actually explains/predicts the target condition *vis a vis* the comparator

   ☐ All models included are generative (i.e. capable of simulating data)

   ☐ Quality of model fit quantified statistically (with respect to empirical data)

   ☐ Quality of model fits compared statistically between models (e.g. Bayesian Model Selection)

   ☐ Model fit and comparisons were done with adequate statistical power

   ☐ Proposed model is best fit for features of the target state/condition

   ☐ Proposed model is not best fit for features of comparator (unless model is modified through intervention; see predictive validity)

   ☐ Best fitting model does not generate additional features that are absent in the target state/condition

2.2 Subscale 2: Predictive Validity

Definition: The degree to which manipulations of the model predict the effects of real world interventions on the target condition of interest. In other words, do interventions on the model result in changes of its behaviour similar to those of an analogous intervention on the real-world target condition? For example, if model M_1 captures depression (D), and model M_2 captures normal mood (euthymia, E), then some transformation $T(M_1, M_2)$ should be a good explanation for scenarios in which one observes $D \to E$.

1. There are identifiable and meaningful transitions between conditions/states of interest in the real-world phenomenon

   ☐ States and transition are explicitly identified (e.g. depression $\to$ euthymia)

- ☐ Nature by which transition occurs in reality is explicitly identified (e.g. medication intervention causing depression to transition into euthymia)
- ☐ Nature of transition in reality is empirically or theoretically based, with appropriate citation
- ☐ Relevance of transition for understanding the condition is explicitly defined (e.g. treatment effect)
- ☐ Relevance of transition of understanding the condition has appropriate theoretical or empirical grounds, with included citations

2. Interventions/transitions in the model explain or predict corresponding transitions in the condition/state of interest
   - ☐ Distinct models are identified for corresponding states of interest
   - ☐ Individual models explain distinct states exclusively (see face validity)
   - ☐ The intervention resulting in transition between models is explicitly defined
   - ☐ Transformation of one model into the other corresponds to transformation between the target states **OR** Transformation of one model into the other predicts transformation between target states that can be verified empirically
   - ☐ Degree to which transformation between models corresponds to transformation between target states is quantified statistically, and deemed to be the best fit, at least in comparison to a "null" or random transformation **OR** Transformation of one model into the other predicts a transformation that should be observed in between the target states/conditions in the real-world. Prediction is quantitative, empirically verifiable, and includes degree of uncertainty

2.3 Subscale 3: Construct Validity

Definition: The degree of homology between the model and mechanisms that are empirically or theoretically deemed to underlie features of the target condition of interest.

1. There is a real and identifiable or plausible mechanism underlying the target condition/state
   - ☐ Explicitly identified
   - ☐ Empirically or theoretically shown to be involved/associated with target features being explained **OR** Model makes explicit testable predictions about the existence of this underlying mechanism
   - ☐ The model architecture is homologous to the mechanism of interest, at an appropriate level of abstraction
2. Model components are defined and linked to specific components of underlying mechanism
   - ☐ Functional arrangement/connections between model components defined and linked to underlying mechanisms
   - ☐ Multiple models were evaluated with competing mechanisms

3 INTER-RATER RELIABILITY RESULTS

Cohen's κ for each individual paper reviewed is plotted in Supplemental Figure 5.

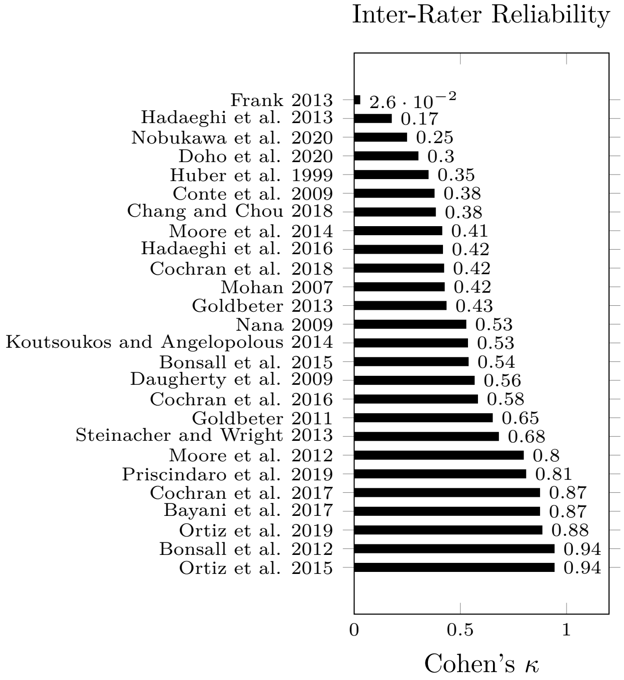

Supplemental Figure 5: Cohen's κ statistics for each of the papers reviewed. Mean κ was 0.55(95% CI [0.45, 0.64]).

4 VALIDITY APPRAISAL GUIDE RESULTS TABLE

Complete results for raters' evaluations of papers using the Validity Appraisal Guide for Computational Models are shown in Supplemental Table 1.

Supplemental Table 1: Complete validity appraisal checklist results after consensus.

	STUDIES																									
	[1]	[3]	[4]	[2]	[5]	[12]	[13]	[11]	[14]	[6]	[10]	[15]	[7]	[16]	[17]	[18]	[19]	[20]	[21]	[22]	[23]	[24]	[25]	[26]	[27]	[28]
**FACE VALIDITY**																										
The model aims to describe a real-world phenomenon (i.e. a target state/condition vis a vis comparators)																										
Target condition defined clearly	1	1	1	1	1	1	1	1	1	1	1	1	1	1	1	1	1	1	1	1	1	1	1	1	1	1
Comparator(s) defined clearly	1	1	1	1	1	0	1	1	0	1	1	0	0	1	0	1	1	0	1	0	1	0	1	1	0	0
The target state/condition being modeled is identifiable according to observable features																										
Features are explicitly defined	1	1	1	1	1	1	1	0	1	0	1	0	1	1	1	0	0	1	1	1	1	1	1	0	1	1
Feature definitions are operationalized	0	0	1	0	0	0	1	0	0	0	1	0	1	1	1	0	0	0	0	1	1	1	0	0	1	1
Relevance of features to condition of interest is stated (i.e. features actually present clinically)	1	0	1	0	0	1	1	0	1	0	1	0	1	1	1	0	0	0	0	1	1	1	0	0	1	1
Features are identified based on cited empirical observations or experiments	1	0	1	0	0	0	1	0	0	0	1	0	1	1	1	1	0	0	0	1	1	1	0	0	1	1
Multiple features considered	0	0	0	0	0	1	0	0	0	0	1	0	0	1	0	0	0	0	0	1	1	0	0	0	1	1
The comparator state/condition being modeled is identifiable according to observable features																										
Features are explicitly defined	1	1	1	1	0	0	1	0	0	0	1	0	0	1	0	0	0	0	1	0	1	0	1	0	0	0
Feature definitions are operationalized	0	0	0	0	0	0	1	0	0	0	1	0	0	1	0	0	0	0	0	0	1	0	0	0	0	0
Relevance of features to condition of interest is stated (i.e. features actually present clinically)	1	0	1	0	0	0	1	0	0	0	1	0	0	1	0	0	0	0	0	0	1	0	0	0	0	0
Features are identified based on cited empirical observations or experiments	1	0	1	0	0	0	1	0	0	0	1	0	0	1	0	1	0	0	0	0	1	0	0	0	0	0
Multiple features considered	0	0	0	0	0	0	0	0	0	0	1	0	0	1	0	0	0	0	0	0	1	0	0	0	0	0
Relationship to features of target condition identified (e.g. mutual exclusivity, correlation, etc.)	1	0	1	1	0	0	1	0	0	0	1	0	0	1	0	0	0	0	1	0	1	0	0	0	0	0
The model actually explains/predicts the target condition vis a vis the comparator																										
All models included are generative (i.e. capable of simulating data)	1	1	1	1	1	1	1	1	1	1	1	1	1	1	1	1	1	1	1	1	1	1	1	1	1	1
Quality of model fit quantified statistically (with respect to empirical data)	0	0	0	0	0	0	1	0	0	0	0	0	1	0	1	0	0	0	0	1	1	1	0	0	1	1
Quality of model fits compared statistically between models (e.g. Bayesian Model Selection)	0	0	0	0	0	0	1	0	0	0	0	0	1	0	1	0	0	0	0	1	0	1	0	0	1	1
Model fit and comparisons were done with adequate statistical power	0	0	0	0	0	0	0	0	0	0	0	0	0	0	0	0	0	0	0	1	1	1	0	0	0	0
Proposed model is best fit for features of the target state/condition	0	0	1	0	0	0	0	0	0	0	1	0	0	0	0	0	0	0	0	1	0	1	0	0	0	0
Proposed model is not best fit for features of comparator (unless model is modified through intervention; see predictive validity)	0	0	1	0	0	0	0	0	1	0	1	0	0	0	0	0	0	0	0	0	0	0	0	0	0	0
Best fitting model does not generate additional features that are absent in the target state/condition	0	0	0	0	0	0	0	0	0	0	0	0	0	0	0	0	0	0	0	0	0	0	0	0	0	0
**PREDICTIVE VALIDITY**																										
There are identifiable and meaningful transitions between conditions/states of interest in the real-world phenomenon																										
States and transition are explicitly identified (e.g. depression $\rightarrow$ euthymia)	1	1	1	1	1	1	0	0	0	1	1	0	0	0	0	0	0	0	1	0	0	0	0	0	0	0
Nature by which transition occurs in reality is explicitly identified (e.g. medication intervention causing depression to transition into euthymia)	1	1	1	1	1	1	0	0	0	1	1	0	0	0	0	0	0	0	1	0	0	0	0	0	0	0
Nature of transition in reality is empirically or theoretically based, with appropriate citation	1	0	0	0	0	1	0	0	0	0	1	0	0	0	0	0	0	0	0	0	0	0	0	0	0	0
Relevance of transition for understanding the condition is explicitly defined (e.g. treatment effect)	1	1	1	1	1	1	0	0	0	0	1	0	0	0	0	0	0	0	1	0	0	0	0	0	0	0
Relevance of transition of understanding the condition has appropriate theoretical or empirical grounds, with included citations	1	0	0	0	0	1	0	0	0	0	1	0	0	0	0	0	0	0	0	0	0	0	0	0	0	0
Interventions/transitions in the model explain or predict corresponding transitions in the condition/state of interest																										
Distinct models are identified for corresponding states of interest	1	1	0	1	1	1	0	1	0	0	1	0	0	0	0	0	0	0	1	0	0	0	0	0	0	0
Individual models explain distinct states exclusively (see face validity)	0	0	0	0	1	1	0	1	0	0	1	0	0	0	0	0	0	0	0	0	0	0	0	0	0	0
The intervention resulting in transition between models is explicitly defined	1	0	0	1	1	1	0	0	0	0	1	0	0	0	0	0	0	0	1	0	0	0	0	0	0	0
Transformation of one model into the other corresponds to transformation between the target states OR Transformation of one model into the other predicts transformation between target states that can be verified empirically	1	1	0	0	1	1	0	0	0	1	1	0	0	0	0	0	0	0	0	0	0	0	0	0	0	0
Degree to which transformation between models corresponds to transformation between target states is quantified statistically, and deemed to be the best fit, at least in comparison to a "null" or random transformation OR Transformation of one model into the other predicts a transformation that should be observed in between the target states/conditions in the real-world. Prediction is quantitative, empirically verifiable, and includes degree of uncertainty	0	0	0	0	0	0	0	0	0	0	0	0	0	0	0	0	0	0	0	0	0	0	0	0	0	0
**CONSTRUCT VALIDITY**																										
There is a real and identifiable or plausible mechanism underlying the target condition/state																										
Explicitly identified	1	0	0	0	0	0	0	1	0	0	0	0	0	0	0	1	0	0	1	0	0	0	0	1	0	0
Empirically or theoretically shown to be involved/associated with target features being explained OR Model makes explicit testable predictions about the existence of this underlying mechanism	1	0	0	0	0	0	0	0	0	0	0	0	0	0	0	0	0	0	1	0	0	0	0	0	0	0
The model architecture is homologous to the mechanism of interest, at an appropriate level of abstraction																										
Model components are defined and linked to specific components of underlying mechanism	0	0	0	0	0	0	0	1	0	0	0	0	0	0	0	1	0	0	0	0	0	0	0	0	0	0
Functional arrangement/connections between model components defined and linked to underlying mechanisms	0	0	0	0	0	0	0	1	0	0	0	0	0	0	0	0	0	0	0	0	0	0	0	0	0	0
Multiple models were evaluated with competing mechanisms	0	1	1	1	0	1	0	0	1	0	0	0	1	1	0	0	1	1	0	1	0	0	0	0	1	0

REFERENCES

[1] M. T. Huber, H. A. Braun, and J. C. Krieg, Biological Psychiatry **46**, 256 (1999).

[2] D. Daugherty, T. Roque-Urrea, J. Urrea-Roque, J. Troyer, S. Wirkus, and M. A. Porter, Communications in Nonlinear Science and Numerical Simulation **14**, 2897 (2009), arXiv:0311032 [nlin] .

[3] M. A. Mohan, in *Conference Proceedings - IEEE SOUTHEASTCON* (2007) pp. 279–282.

[4] E. Conte, A. Federici, G. Pierri, L. Mendolicchio, and J. P. Zbilut, in *Progress in Chaos and Complexity Research* (Nova Research, New York, NY, 2009) pp. 25–44.

[5] L. Nana, Communications in Nonlinear Science and Numerical Simulation **14**, 351 (2009).

[6] F. Hadaeghi, M. R. Hashemi Golpayegani, and S. Gharibzadeh, Frontiers in Computational Neuroscience (2013), 10.3389/fncom.2013.00106.

[7] M. B. Bonsall, J. R. Geddes, G. M. Goodwin, and E. A. Holmes, Journal of The Royal Society Interface **12**, 20150670 (2015).

[8] B. van der Pol, Radio Review (London) **1**, 701 (1920).

[9] B. van der Pol, The London, Edinburgh, and Dublin Philosophical Magazine and Journal of Science **2**, 978 (1926).

[10] A. Steinacher and K. A. Wright, PLoS ONE **8** (2013), 10.1371/journal.pone.0063345.

[11] T. D. Frank, Communications in Nonlinear Science and Numerical Simulation **18**, 2107 (2013).

[12] A. Goldbeter, Progress in Biophysics and Molecular Biology **105**, 119 (2011).

[13] M. B. Bonsall, S. M. A. Wallace-Hadrill, J. R. Geddes, G. M. Goodwin, and E. A. Holmes, Proceedings of the Royal Society B: Biological Sciences **279**, 916 (2012), publisher: Royal Society.

[14] A. Goldbeter, Pharmacopsychiatry **46 Suppl 1**, 44 (2013).

[15] E. Koutsoukos and E. Angelopoulos, International Journal of Bipolar Disorders **2**, 1 (2014).

[16] A. Ortiz, K. Bradler, J. Garnham, C. Slaney, and M. Alda, Bipolar Disorders **17**, 139 (2015).

[17] A. L. Cochran, M. G. McInnis, and D. B. Forger, Translational Psychiatry **6**, e912 (2016).

[18] F. Hadaeghi, M. R. Hashemi Golpayegani, S. Jafari, and G. Murray, Australian and New Zealand Journal of Psychiatry **50**, 783 (2016).

[19] A. Bayani, F. Hadaeghi, S. Jafari, and G. Murray, Chronobiology International **34**, 235 (2017).

[20] A. L. Cochran, A. Schultz, M. G. McInnis, and D. B. Forger, in *Computational Neurology and Psychiatry*, Vol. 6, edited by P. Érdi, B. Sen Bhattacharya, and A. L. Cochran (Springer International Publishing, Cham, 2017) pp. 315–341, series Title: Springer Series in Bio-/Neuroinformatics.

[21] S.-S. Chang and T. Chou, Computational Psychiatry **2**, 205 (2018).

[22] A. L. Cochran, A. Schultz, M. G. McInnis, and D. B. Forger, Translational Psychiatry **8**, 36 (2018).

[23] A. Ortiz, K. Bradler, J. Garnham, C. Slaney, S. McLean, and M. Alda, Journal of Affective Disorders **243**, 274 (2019).

[24] J. J. Prisciandaro, B. K. Tolliver, and S. M. DeSantis, Psychological Medicine **49**, 1102 (2019).

[25] H. Doho, S. Nobukawa, H. Nishimura, N. Wagatsuma, and T. Takahashi, Frontiers in Computational Neuroscience **14**, 76 (2020).

[26] S. Nobukawa, H. Nishimura, H. Doho, and T. Takahashi, Frontiers in Applied Mathematics and Statistics **6**, 53 (2020).

[27] P. J. Moore, M. A. Little, P. E. McSharry, J. R. Geddes, and G. M. Goodwin, IEEE Transactions on Biomedical Engineering **59**, 2801 (2012).

[28] P. J. Moore, M. A. Little, P. E. McSharry, G. M. Goodwin, and J. R. Geddes, International Journal of Bipolar Disorders **2**, 1 (2014).
